# Primary endobronchial multifocal Ewing’s sarcoma: a rare case report

**DOI:** 10.3389/fonc.2024.1431950

**Published:** 2024-08-30

**Authors:** Dan Liu, Xiaoge Liu, Xin Li, Yisha Liu, Junlun Yu

**Affiliations:** ^1^ Department of Radiology, Sichuan Provincial People’s Hospital, University of Electronic Science and Technology of China, Chengdu, China; ^2^ Department of Ultrasound, Ya’an People’s Hospital, Ya’an, China; ^3^ Department of Pathology, Sichuan Provincial People’s Hospital, University of Electronic Science and Technology of China, Chengdu, China

**Keywords:** Ewing’s sarcoma, endobronchus, thoracic tumor, EWSR1-FLI1 fusion gene, case report

## Abstract

Extraskeletal Ewing’s sarcoma (ES) has been reported to originate from various sites. Primary endobronchial ES is an extremely rare bronchial tumor, especially multifocal lesions. This report describes a rare presentation of primary bronchial ES in a 31-year-old female who was referred to the emergency department of our hospital due to suspicion of a foreign body in the bronchus. Computed tomography and bronchoscopy revealed multiple polypoid nodules in the middle bronchus of her right lung, thus excluding the initial diagnosis. Infection-related laboratory tests and serum tumor markers were normal. The bronchial sleeve resection was performed to remove the tumor completely and the patient’s clinical symptoms obviously improved. Subsequent imaging, histopathological, immunohistochemical and genetic analyses made a conclusive diagnosis of primary endobronchial ES. To our knowledge, this is the eighth case of primary bronchial ES reported in medical literature.

## Introduction

The Ewing sarcoma family of tumors (ESFT) is a group of small round cell neoplasms that have similar neuroectodermal origin and chromosomal translocation (1).While the vast majority of ESFT cases occur in children and in bones, approximately 15%-20% originate outside the skeleton as extraskeletal Ewing’s sarcoma (EES) (2). EES was first described by Angervall and Enzinger (3) in 1975. EES typically originates from the deep soft tissues of the trunk, extremities, retroperitoneum, and chest wall. EES has also been reported in the kidneys, adrenal glands, pancreas, uterus, gastrointestinal tract, and other visceral organs (4). EES that arises in the bronchus are extremely rare, especially multifocal lesions. To our knowledge, primary endobronchial multifocal ES in the middle bronchus of the right lung has not previously been reported. This case report describes a rare primary endobronchial ES in the middle bronchus of the right lung in a 31-year-old female. The patient underwent thoracic surgical exploration and sleeve bronchial resection, and preoperative imaging studies, postoperative pathological results and genetic analysis confirmed the diagnosis of primary bronchial ES.

## Case report

**Figure 1:**
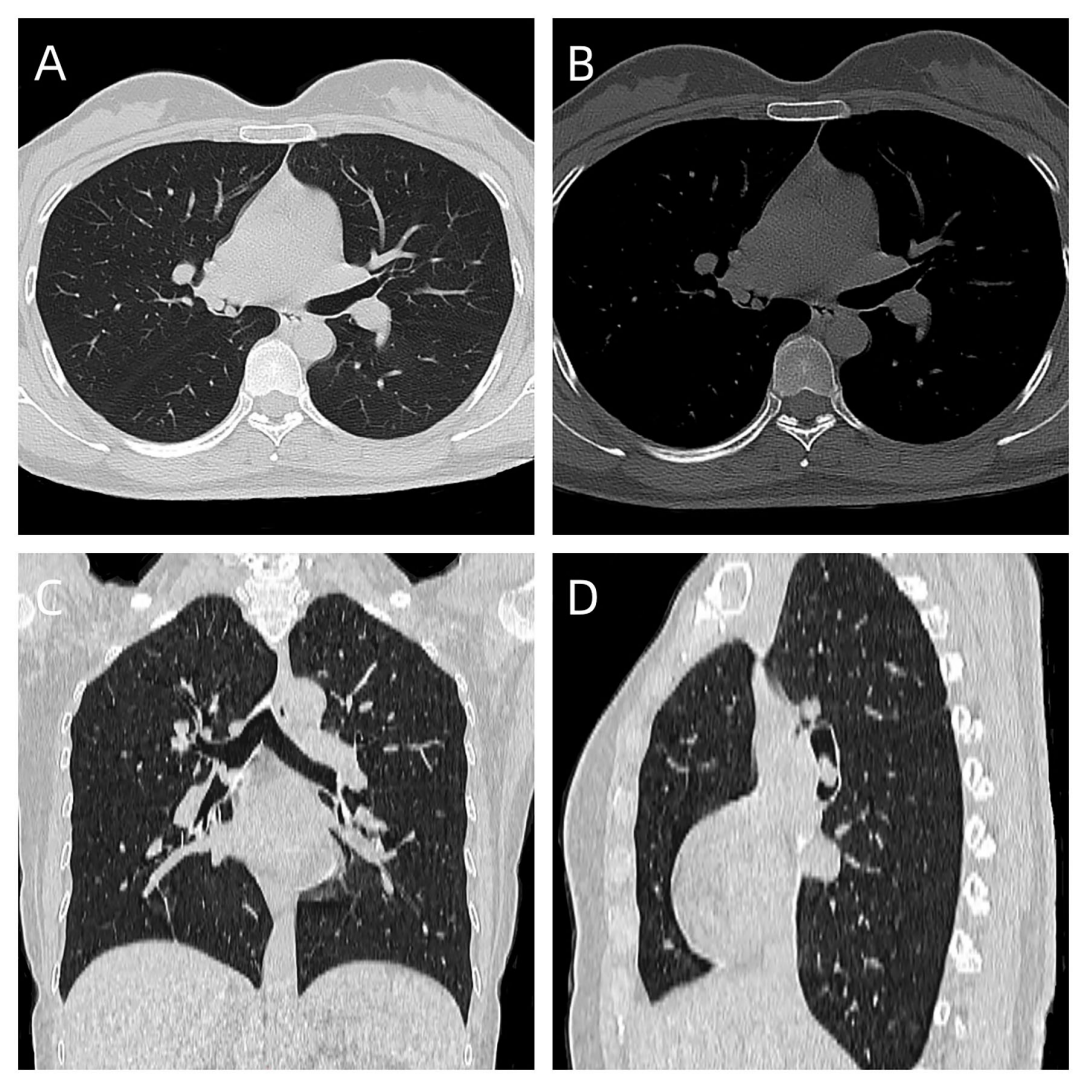
**(A)** axial lung window, CT showed multiple polypoid lesions in the middle bronchus of the right lung; **(B)** axial bone window; **(C)** coronal scan; **(D)** saggital scan.

**Figure 2:**
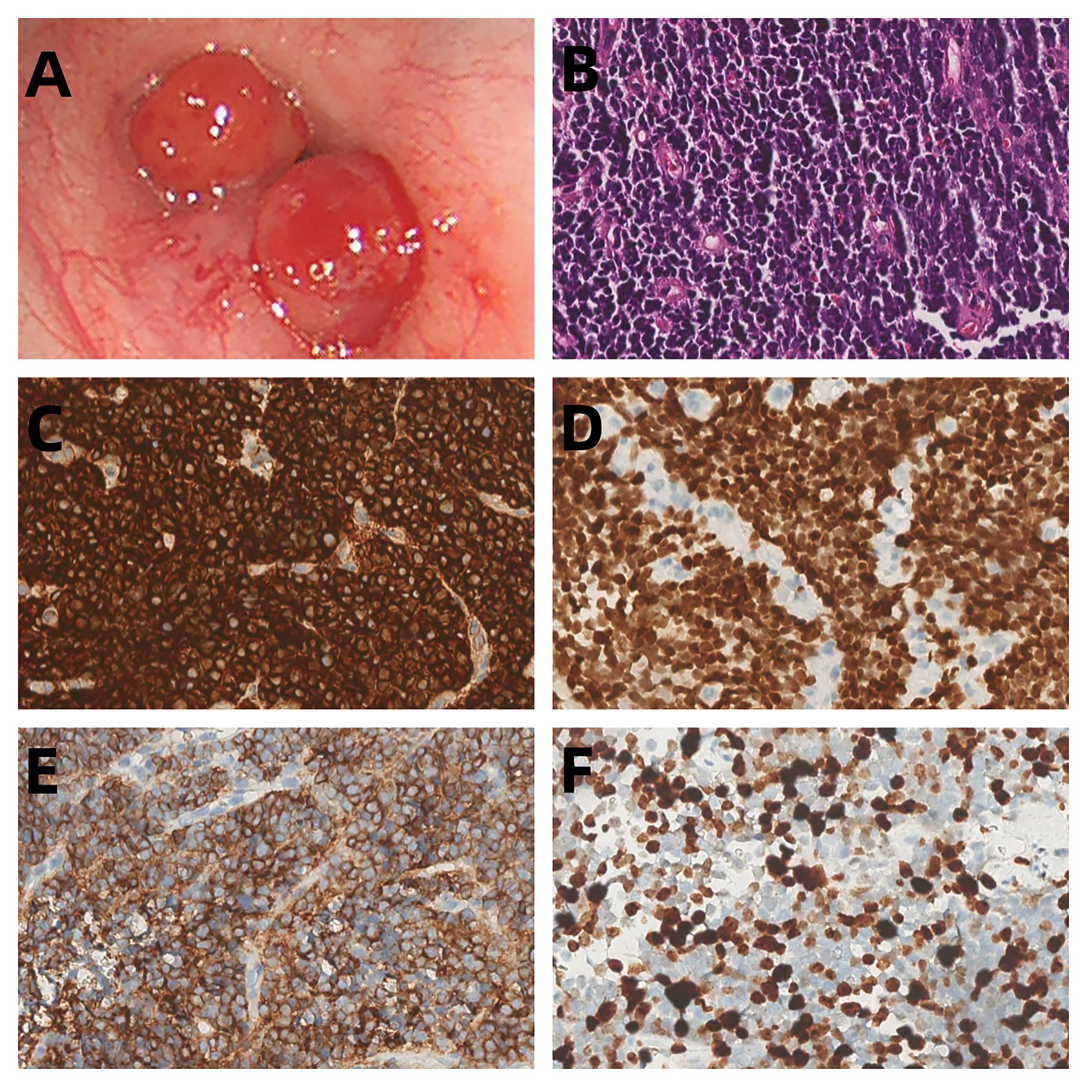
**(A)** Bronchoscopic images demonstrated polypoid endobronchial tumors that originated in the middle bronchus of the right lung. **(B)** The tumor cells show small, round-to-oval nuclei without rosette-like arrangement (HE, original magnification 200). **(C–E)** Immunohistochemical findings showed strongly positivity for CD99, NKX2.2 and CD56 **(F)** The Ki-67 labeling index was approximately 50%.

A 31-year-old female presented with a one-week history of slight pharyngeal discomfort and choking cough while lying supine. She had an occasional audible whistle when she spoke loudly. Her symptoms worsened after eating fish with black beans 3 days previously. Computed tomography (CT) performed at another hospital detected multiple polypoid lesions in the middle bronchus of the right lung, the largest measuring approximately 0.7 cm×0.7 cm×0.6 cm (**Figure 1**). The tumors exhibited well-defined borders and homogeneous density. The walls of bronchi were smooth. She was admitted to the emergency department of our hospital due to suspicion of a foreign body in the bronchi. The initial diagnosis was ruled out after scanning the CT images. Tuberculosis was suspected based on the patient’s age. Infection-related laboratory tests and serum tumor markers were normal. She denied any prior history of fever, recent chest trauma, contact with tuberculosis, or exposure to toxins. Her family denied a history of malignancy. The vital signs were stable, the thoracic contour was normal; there were no dry or moist rales and pleural friction sounds. More prevalent low-grade malignant tumors, such as squamous-cell carcinoma, mucoepidermoid carcinoma, and carcinoid tumor, which prompted bronchoscopic biopsy, were also doubted. Bronchoscopy revealed grey-white polypoid masses based on the wall of right middle bronchus. The lesions partially obstructed the middle bronchus with no evidence of invasion (**Figure 2A**). Transbronchial needle aspiration was performed and histological results revealed typical small blue tumor cells without necrosis and then the patient was referred to West China Hospital for further treatment. Right bronchial sleeve resection was performed, and polypoid endobronchial nodules were observed, the largest measuring approximately 1.0 cm×1.0 cm×1.0 cm. Grossly, the tumors appeared to be *in situ*, however, microscopically, they infiltrated the submucosa, muscles, and cartilages. No perineural, vascular, or lymphatic invasion was observed. Hematoxylin and eosin (H&E) staining revealed monomorphic population of small blue cells with variably conspicuous nucleoli and scant cytoplasm (**Figure 2B**). Immunohistochemistry analysis demonstrated strong positivity for CD99, NKX2.2, CD56, P63, INI-1, and negativity for PCK, Syn, CgA, EMA, DSE, Myogenin, S100, SMA, WT-1, LCA, CK8/18 (**Figures 2C–E**). The Ki-67 labeling index was approximately 50% (**Figure 2F**). Fluorescence *in situ* hybridization (FISH) for *EWSR1* gene translocation was positive, and next-generation sequencing confirmed *EWSR1-FLI1* gene fusion. ECT, MRI of the head and neck and multisite ultrasonography results were normal. Therefore, this tumor was eventually identified as a primary endobronchial ES. The patient underwent 17 courses of chemotherapy after resection including 2 alternating blocks: VDC (vincristine 2 mg/m2, doxorubicin 60 mg/m2, cyclophosphamide 200 mg/m2, respectively, on day 1, every 4 weeks) and IE (ifosfamide 2600 mg/m2, etoposide 150 mg/m2, respectively, on days 1 to 5, every 4 weeks). The radiotherapy was not performed. Chest CT, abdominal MRI, and bronchoscopy were performed annually. At the 48-month follow-up after the operation, the patient remained disease-free.

## Discussion

ES is a highly aggressive malignancy with poor prognosis (5) that usually affects the bones, particularly the femur, pelvis or axial skeleton. It is most commonly diagnosed in the second decade of life, with a slight male predominance (6). The majority of patients with EES are young, with a median age of 20 years, they are 5–10 years older than those with ES of the bone (ESB); the age range is also much wider. Compared with ESB, EES demonstrates a less strong predilection for male patients, with a more even distribution among men and women (7). For bronchial ES, including our case (a 31-year-old female), reports in the literature describe the details of 6 males and 2 females aged 12-65 years (mean age 29.5 years). Our findings are consistent with those of previous reports.

EES has been reported in the thorax, extremities, head and neck, pelvis, abdomen, spine, intracranial space, orbit and so on (8). Bronchial ES is extremely rare, with only 7 cases reported in the literature, and our patient is the eighth such documented case of primary bronchial ES (**Table 1**) (9–15). Moreover, to the best of our knowledge, an ESS originating in the right lung’s middle bronchus has never been reported. Due to the low incidence and atypical clinical manifestations of EES, accurate diagnosis, differential diagnosis, and subsequent treatment options are challenging.

**Table 1:** Case reports of primary bronchial Ewing sarcoma published in the literature.

Case No.	Authors	Year published	Age/Gender	Location	Size (mm)	Surgery	Treatment	Follow up
1	Kahn et al (9)	2001	18/M	Right middle lobe bronchus	40	Lobectomy	None	24 months, local recurrence and death
2	Tane et al (10)	2012	12/M	Right main bronchus	18	Bronchoscopic resection	Adjuvant chemoradiation	12 months, no recurrence
3	Hayakawa et al (11)	2013	12/M	Right main bronchus	20	Bronchoscopic resection	Adjuvant chemoradiation	18 months, no recurrence
4	Han et al (12)	2015	29/M	Left main bronchus	40	Left pneumonectomy	None	18 months, no recurrence
5	Chen et al (13)	2018	30/M	Left lower lobe bronchus	64	Left lower lobectomy	Adjuvant chemotherapy	18 months, no recurrence
6	Mumtaz et al (14)	2019	64/M	Left main bronchus	16	Sleeve bronchial resection	None	9 months, no recurrence
7	Kirishima et al (15)	2021	39/F	Right lower lobe bronchus	18	Right lower lobectomy	None	24 months, no recurrence
8	Our case	2024	31/F	Right middle bronchus	10	Sleeve bronchial resection	Adjuvant chemotherapy	48 months, no recurrence

The clinical symptoms vary depending on the region of involvement. Symptoms include irritable cough (87.5%), dyspnea (62.5%), fever (25%), hemoptysis (12.5%), chest pain (12.5%), and pharyngeal discomfort(12.5%). These clinical manifestations of bronchial ES are atypical and may be easily mistaken for other bronchial tumors or diseases. As in our case, the patient initially complained of some throat irritation and a choking cough. After consuming fish with black beans, the patient’s symptoms worsened, and she was mistaken for a body foreign in her bronchus. Subsequent comprehensive analyses led to the definitive diagnosis of primary endobronchial ES. It should be distinguished from squamous cell carcinoma, cartinoid tumor, mucoepidermoid carcinoma, adenoid cystic carcinoma, small cell carcinoma, adenocarcinoma, lymphoma rhabdomyosarcoma and leiomyosarcoma (16).

Chest radiography may provide less information for primary bronchial ES due to shading of the mediastinal structures. However, direct radiographic evidence, such as a pulmonary hilar mass and indirect manifestations, such as obstructive atelectasis and obstructive pneumonia may still be found, if the tumor has grown to a considerable size or into the lumen, even in a small volume. In our study, 6 of 8 patients underwent this examination; pulmonary hilar mass was observed in 3 (37.5%); obstructive atelectasis in 4 (50%) and pneumonia in 2 (25%).

Chest CT, particularly three-dimensional imaging, offers superior clarity in visualizing intrabronchial tumors and can assist in the early suggestive diagnosis of primary bronchial ES (17). In our study and the literature, 5 of 8 (62.5%) were located in the right bronchus, and the remaining 3 (37.5%) were found in the left bronchus. Of these cases, 5 (62.5%) invaded the bronchial wall with or without extension to the pulmonary parenchyma; these tumors presented with irregular shape, ill-defined borders, and homogeneous or heterogeneous density (average diameter, 32mm).The remaining 3 (37.5%) were confined to the bronchial lumen, showing nodular shape, smooth, and well-defined margins with homogeneous density (average diameter, 22.7mm). Three of 8 patients underwent CT-enhanced scanning, in which 1 of 3 tumors (33.3%) exhibited homogeneous enhancement, while 2 (66.7%) exhibited multiple focal heterogeneous enhancements. Furthermore, we discovered multifocal tumors in our case, a finding that has not been previously reported in the literature. The etiology of this phenomenon remains unknown.

Currently, ES diagnosis depends mainly on the comprehensive histopathological, immunohistochemical, and genetic analyses.

Histopathologically, ES is characterized by an abundance of small round blue cells arranged in compact sheets with a low amount of eosinophilic cytoplasm and a high nuclear-to-cytoplasmic volume ratio (18). ES must be differentiated from malignant lymphoma, neuroblastoma, and embryonal rhabdomyosarcoma (19). The differentiation of ES from the other entities may occasionally be difficult, especially in the soft tissue variants. The individual cells in ES are round, of moderate size, have clear and frequently quite scant cytoplasm, and round to oval nucleus. Classical ES cells are frequently positive for glycogen. The final diagnosis depends on the immunohistochemical and genetics findings.

ES is driven by a non-random chromosomal translocation events involving the EWSR1 gene located on chromosome 22, and members of the ETS family of transcription factors, most commonly FLI1 on chromosome 11 (85%), Other fusion partners include ERG (10%), ETV1 (<1%), and ETV4 (<1%) and so on (20). In our study, genetic testing was performed in 4 of the 8 cases of bronchial ES, and the *EWSR1-FLI-1* fusion formed by t (11, 21)(q24;12) chromosomal translocation was found in 3 patients, including our case, while the results in other patients were negative.

CD99 (the product of the MIC2 antigen) represents the most useful molecular marker for ES diagnosis, although its diagnostic specificity and sensitivity for ES are not 100% (21). Strong CD99 membrane immunopositivity is seen in practically all examples of ES. In our study, CD99 was found to be strongly positive in all 8 reported cases. Due to its remarkable sensitivity, CD99 immunonegativity would strongly argue against a diagnosis of ES. FLI1 and ERG immunopositivity can be seen in those ES harboring *EWSR1-FLI1* (22) and *EWSR1-ERG* gene fusions (23), respectively. NKX2.2, a homeodomain transcription factor involved in neuroendocrine/glial differentiation and a downstream target of *EWSR1-FLI1*, is recognized as a specific molecular marker in the diagnosis of ES, and its combination with CD99 makes this test highly specific (24). In our study, we observed 1 case each with positive FLI-1 and NKX2.2 expression. In addition, we detected vimentin(3/8), synaptophysin (3/8), cytokeratin (2/8), P63 (2/8) and CD56 (2/8). However, the diagnostic utility of these markers requires further investigation.

ES is generally difficult to treat. Patients frequently relapse and require complex treatment regimens including surgery, radiotherapy, and chemotherapy (25). Owing to the rarity of bronchial ES tumor, discrepancies in their clinical presentation and differences in patient characteristics, the optimal management of bronchial ES tumors remains unclear (26).

For bronchial ES tumors, surgical resection with negative margins, when possible, appears to offer the best chance for disease-free survival compared with additional use of neoadjuvant or adjuvant therapy. The treatment and prognosis of 8 bronchial ES tumors reported in the literature is summarized in **Table 1**. Eight patients all underwent different surgical procedures with or without adjuvant therapy, only 1 of whom experienced recurrence and death. This result suggests that although the bronchial ES is an aggressive tumor, the prognosis was better than those originating in other organs.

The surgical strategy for bronchial ES is contingent on the specific bronchial segment affected. Tumors in the distal bronchial airway and pulmonary parenchyma may requires lobectomy to maintain negative margins. For tumors located proximally in the bronchus, bronchoscopic snare resection may be an option. However, this method increases the risk of local recurrence because bronchial ES tumors usually present as pedunculated, exophytic tumors which can spread into the submucosal layer, muscle, and cartilage (12). In the present case, preoperative CT and bronchoscopy identified multiple polypoid lesions characterized by smooth adjacent bronchial walls. Macroscopically, these tumors appeared to be confined in the bronchial wall; however, microscopic evaluation revealed infiltration into the submucosa, muscle layers, and cartilages. Bronchial sleeve resection is a surgical option to remove the tumor completely and preserves as much healthy lung tissue as possible.

Prognosis depends on stage, tumor location and size, patient age, and response to chemotherapy. The multimodal approach to the treatment of ES has allowed the 5-year survival rate for localized tumors to be 70% to 80%. EES tends to have a better prognosis than osseous ES (27). Our patient, a 31-year-old female, is the second reported case of primary bronchial ES treated safely by sleeve resection with chemotherapy, and the patient was alive with no evidence of recurrence on imaging scans and bronchoscopy at 48 month’s follow up.

Although rare, we should consider the possibility of bronchial EES when we are evaluating the bronchial lesions. Further in-depth research and accumulation of data from similar cases are essential to refine our understanding and management strategies for bronchial ES.

## Conclusion

Our case highlights the complex diagnosis and management of primary bronchial ES, an uncommon and highly aggressive malignancy with an atypical presentation. We aimed to expand the existing knowledge on this unusual entity and promote further research to enhance therapeutic strategies for primary bronchial ES. Furthermore, it underscores the indispensable significance of an interdisciplinary approach in handling rare and intricate cases of thoracic surgery and oncology.

## Data Availability

The original contributions presented in the study are included in the article/supplementary material. Further inquiries can be directed to the corresponding author.
